# Molecular Biology of Rotavirus Entry and Replication

**DOI:** 10.1100/tsw.2009.158

**Published:** 2009-12-16

**Authors:** Marie Christine Ruiz, Theresa Leon, Yuleima Diaz, Fabian Michelangeli

**Affiliations:** Laboratorio de Fisiología Gastrointestinal, Instituto Venezolano de Investigaciones Cientícas (IVIC), Caracas 20632, Venezuela

**Keywords:** rotavirus entry, receptors, structure, endocytosis, replication, assembly, Ca^2+^ compartments, Ca^2+^ homeostasis, structural viral protein, nonstructural viral protein, viroplasm, endoplasmic reticulum, viroporin

## Abstract

Rotavirus is a nonenveloped, double-stranded, RNA virus belonging to the Reoviridae family and is the major etiological agent of viral gastroenteritis in young children and young animals. Remarkable progress in the understanding of the rotavirus cycle has been made in the last 10 years. The knowledge of viral replication thus far acquired is based on structural studies, the expression and coexpression of individual viral proteins, silencing of individual genes by siRNAs, and the effects that these manipulations have on the physiology of the infected cell. The functions of the individual rotavirus proteins have been largely dissected; however, the interactions between them and with cell proteins, and the molecular mechanisms of virus replication, are just beginning to be understood. These advancements represent the basis for the development of effective vaccination and rational therapeutic strategies to combat rotavirus infection and diarrhea syndromes. In this paper, we review and try to integrate the new knowledge about rotavirus entry, replication, and assembly, and pose some of the questions that remain to be solved.

### INTRODUCTION

Viral infections are responsible for a high degree of morbidity and mortality around the world. Thorough knowledge of the mechanisms of virus spread, target entry, replication strategy, and pathogenesis are quintessential to the control of the disease. A number of animal virus infections result in lysis of the host cell, which is usually due to disturbances in cell function induced by viral genome expression and viral protein synthesis. A progressive change in membrane permeability to both monovalent and divalent cations, and then to macromolecules of increasing size, is observed. These changes are linked to a disruption of membrane integrity preceding cell death. The nature of the cell membrane modifications at the molecular level and the identity of the viral products responsible for these modifications are fundamental aspects in understanding the mechanisms of cell permeabilization and lysis by viral infections[1].

Finely, synchronized changes in the concentration of $Ca^{2+}$ ions in the cytoplasm ($[Ca^{2+}]_{cyto}$) modulate a variety of intracellular functions both in physiological conditions and pathological states. Intracellular

$[Ca^{2+}]$ is regulated by interactions among transporters, pumps, channels, and binding proteins. This is a complex concert of events that requires the intervention of multiple pathways and mechanisms controlling influx and efflux. Calcium influx into the cytoplasm is due to either $Ca^{2+}$ release from intracellular stores of the endoplasmic reticulum (ER) via IP3 or ryanodine-sensitive channels, or $Ca^{2+}$ influx across the plasma membrane through a variety of $Ca^{2+}$-permeable channels. Calcium efflux from the cytoplasm is the result of $Ca^{2+}$ uptake by $Ca^{2+}$ pumps of the ER (SERCA pump) and other transport mechanisms in other organelles, and extrusion by the $Ca^{2+}$-ATPase and other transporters, such as $Na^+/Ca^{2+}$ exchange located at the plasma membrane[2,3].

Among pathological processes, viral infections depend on disturbances of $Ca^{2+}$ balance. Changes in $Ca^{2+}$ homeostasis of the cells can be observed and $Ca^{2+}$ may act as a messenger in the chain of events that leads to disease[4]. Perhaps the best characterized virus from this point of view is rotavirus and this was previously reviewed[5].

Rotavirus is a nonenveloped, double-stranded, RNA virus belonging to the Reoviridae family and is the major etiological agent of viral gastroenteritis in young children and young animals[6]. These viruses induce acute diarrhea and dehydration, which often require hospitalization. The prevalence of infection is similar in all countries; however, in developing countries, these infections represent an important cause of mortality in young children. Rotaviruses are responsible for around 500,000 deaths per year worldwide. Therefore, development of effective vaccination and therapeutic strategies to combat these viruses is of immediate concern. These require a basic understanding of the molecular mechanisms of virus replication. Two new rotavirus vaccines have recently been licensed that would diminish rotavirus disease among all children. However, vaccine efficacy in developing countries where disease prevention is required remains to be fully evaluated[7].

Rotaviruses primarily infect the mature enterocytes of the tip of the intestinal villi of the jejunum and ileum[8]. Furthermore, it may cause extraintestinal infections[9,10,11]. Pathophysiology of rotavirus diarrhea is the result of many factors where changes in ionic homeostasis lead to dysfunction of the enterocyte, increased secretion, altered motility, cell death, and reduction of the absorptive surface of the intestine and hence malabsorption[8,12,13,14,15]. Disruption of the infected enterocytes may serve also to release virus progeny and products of viral synthesis, and a further amplification of the disease.

Rotavirus infection of the host cell is characterized by a number of $Ca^{2+}$-dependent virus-cell interactions[5]. During the replication cycle, from entry to the release of newly formed particles, the forming virions transit through different cellular compartments, each one characterized by a distinct $[Ca^{2+}]$ that is determinant for the replication process. In this paper, we review various aspects of the rotavirus replication cycle, focusing on the role of $Ca^{2+}$ in entry and assembly of the new particles, and provide new elements on $Ca^{2+}$ homeostasis changes during infection.

### ROTAVIRUS ENTRY

Rotavirus entry into the host cell is a complex multistep process in which different domains of the rotavirus surface proteins interact with different cell surface molecules, which act as putative receptors on the plasma membrane.

#### Rotavirus Structure

The mature, infectious, rotavirus particle, 100 nm in diameter (including the spikes), is an icosahedral, triple-layered particle (TLP) containing a genome formed by 11 segments of double-stranded RNA that code for six structural (VP1, VP2, VP3, VP4, VP6, and VP7) and six nonstructural (NSP1–NSP6) proteins (Fig. 1)[16,17]. The most internal layer of the capsid, formed by the autoassembling VP2, encloses minor proteins (VP1 and VP3) and the genome. The intermediate layer is constituted by VP6 organized in 260 trimers.

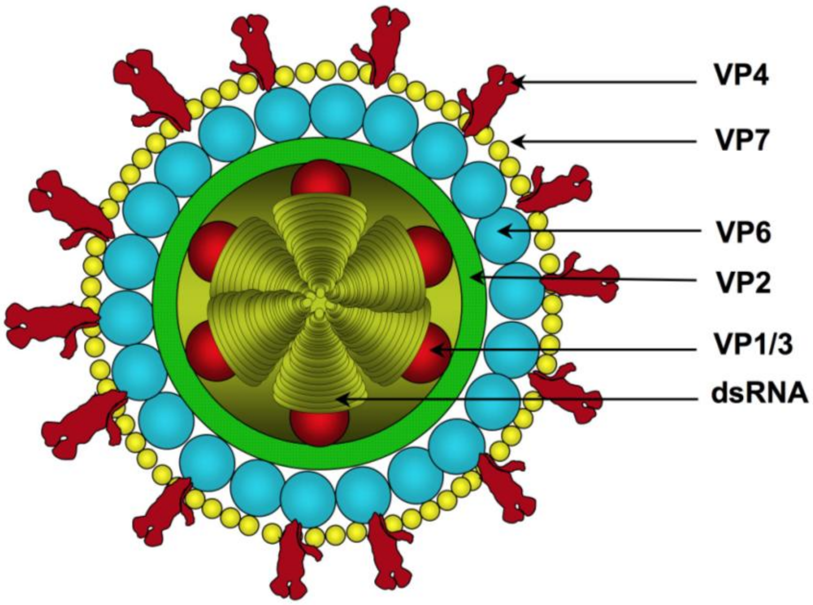

**FIGURE 1**. Schematic depiction of the structure of mature rotavirus particle (TLP). The three concentric capsid protein layers are colored such that red represents VP4 spikes, yellow is the VP7 layer forming the outer layer, blue is the VP6 layer, and green is the VP2 layer. The dsRNA segments (brown) are packed inside the core associated to the RNA polymerase complex (VP1 and VP3, red balls). No inference to stoichiometry is made.

The outer layer contains two proteins, VP7 and VP4, which are involved in the interactions with a putative receptor(s) on the plasma membrane of the host cell and in cell penetration. This layer consists of 260 trimers of the glycoprotein VP7, the major constituent of the layer, and 60 spikes of VP4 that protrude from the VP7 layer[18,19]. Each spike is composed of VP4 dimers; however, a third flexible molecule of VP4 seems to be inserted at its base[17,20,21].

VP4 contains 776 amino acids and has been implicated in viral attachment, neutralization, virulence, host range, and immunity[22,23]. An efficient infectivity of rotavirus in cell culture requires trypsin cleavage of VP4 into two fragments, VP8* (28 kDa, aa 1–247) and VP5* (60 kDa, aa 248–776), both of which remain associated with the virion[24,25]. This hydrolysis seems to occur in physiological conditions, since rotavirus released in the gut has naturally cleaved VP4, probably by endogenous trypsin present in the tract[26]. VP5* contains a hydrophobic region (aa 385–404) that is homologous to the fusogenic region of the glycoprotein of α-viruses[27].

VP4 spikes on virions have “head”, “body”, “stalk”, and “foot” regions, formed by VP8* and VP5*[20,28]. VP8* forms the “head” of the spikes and binds sialic acid in some rotavirus strains. Together with the N-terminus of VP8*, VP5* forms the spike “body”. The body is linked by an asymmetric stalk to a “foot”, which is formed by the carboxyl terminus of VP5* and buried beneath the VP7 shell. The rotavirus particles grown without trypsin have flexible and disordered VP4 spikes. The treatment of these viruses with extracellular trypsin results in only a partial recovery of spike structure and enhancement of infectivity[29]. A second rearrangement of VP4 has been proposed, leading to reorganization where each subunit folds back on itself, translocating a potential membrane-interaction peptide from one end of the spike to the other. This rearrangement may resemble the conformational changes of membrane fusion proteins of enveloped viruses[20].

Calcium stabilizes the outer layer linked to the trimerization of VP7[30,31,32]. Decreasing the concentration of free $Ca^{2+}$ solubilized the outer layer proteins VP4 and VP7 of TLP, generating a double-layer particle (DLP). This treatment greatly reduces the specific infectivity of the virus. All rotavirus serogroups show a similar dependence of rotavirus structure stability on $Ca^{2+}$. The critical concentration of $Ca^{2+}$ for solubilization of the external layer varies from 900 n*M* for the bovine strain RF to 20 n*M* for the simian strain SA11[33]. The use of reassortant rotavirus indicated that this phenotype is linked to VP7. The prolines 279 and 75 appear to be essential for the conformation of the calcium binding site on VP7[34]. The inverse process can be observed *in vitro* where restitution of $Ca^{2+}$ in the medium at acidic pH allows the reassembly of native or recombinant outer shell proteins on the DLP and a significant recovery of specific infectivity, yielding particles as infectious as authentic purified virions[35,36]. VP4 must be added before VP7 to obtain a high level of infectivity[36].

### Rotavirus Receptors

Rotaviruses exhibit cell-specific tropisms consistent with the existence of surface receptor(s) that mediates virus attachment and/or entry through interactions with its external proteins, VP4 and VP7. However, the identity of the rotavirus cellular receptor(s) remains controversial. The sensitivity of rotavirus infection to neuraminidase treatment of the host cell led us to think that sialic acid (SA) residues on the cell surface were required for efficient binding and infectivity[37,38]. However, analysis of a larger number of animal rotavirus strains showed that, like human rotaviruses, most strains are SA independent[39]. Binding of SA-dependent strains to cells seems to be initially mediated by VP8*[40,41], whereas that of SA-independent ones appears to be mediated directly by VP5*[42]. The binding of SA-dependent and SA-independent rotaviruses to glycoconjugates (glycoproteins, glycolipids, and glycosphingolipids) has implicated several molecules as putative rotavirus receptors[43]. Rotavirus VP4 binds to SA with a broad specificity and a low affinity. This is consistent with an initial interaction mediating cell attachment prior to further interactions that would determine host range and cell type specificity[44].

Other types of cell proteins have been implicated in rotavirus entry and may serve as coreceptors or entry factors[45,46,47]. The cellular integrins $\alpha 2\beta 1$, $\alpha 4\beta 1$, $\alpha 4\beta 7$, and hsc70 have been involved in the interaction with VP5*, whereas integrins $\alpha x\beta 2$ and $\alpha v\beta 3$ can bind rotaviruses through the other external protein VP7[48,49,50,51,52,53,54,55,56,57,58,59,60].

### Rotavirus Penetration

After attachment, viruses generally gain access to the cytoplasm by crossing the plasma membrane or the endosomal membrane, in the case of direct entry or endocytosis, respectively. Enveloped viruses can penetrate the cell without disrupting the membrane by fusion of its lipid-containing envelope with either the plasmalemma (e.g., HIV, Sendai, herpes) or the endosomal membrane (e.g., Semliki Forest virus, influenza). In these cases, the binding of the viral membrane protein to the cell receptor, or the acidic medium within the endosome, triggers a conformational change in a viral protein, resulting in the exposition of a fusion region to interact with the target membrane. The mechanisms of entry of naked viruses without a lipid envelope, such as rotavirus, are not well established. Since a fusion process does not really occur, a disruption of the membrane and/or formation of a pore have to be postulated. In the case of rotavirus entry, the TLP must lose its external layer and the large transcriptionally active subviral particle, DLP, be translocated across the plasma or endosomal membrane into the cytoplasm of the target cell. How and where VP4 and VP7 mediate TLP uncoating and DLP entry remain controversial.

#### *Entry Pathways*

Two pathways have been proposed for rotavirus entry into the host cell: direct penetration through the cell membrane and receptor-mediated endocytosis.

Electron microscopy studies do not permit us to distinguish between the two pathways. Numerous ultrastructural observations reveal images of rotavirus particles within coated pits, coated vesicles, and endosomes during entry[61,62,63,64]. Also, images suggestive of direct entry have been described[64]. However, the difficulty stands in assessing which of these lead to productive penetration[65,66].

The strongest evidence in favor of a direct entry is the observation of a change of membrane permeability during rotavirus entry measured as an efflux of intracellular-space markers from prelabeled MA104 cells[66]. This result has been interpreted as the reversible destabilization of the membrane during direct entry through the plasma membrane. The capacity of rotavirus particles to induce from without the formation of syncytia between cells has also been used as an argument in favor of the direct route, since syncytium formation and rotavirus penetration shared similar requirements[67,68,69]. However, these two processes may not be necessarily related.

General metabolic inhibitors (sodium azide, dinitrophenol) and lysosomotropic agents (ammonium chloride, chloroquine) had little effect on the entry of infectious virus into cells, suggesting that the entry of rotavirus is independent of low endosomal pH[61,66,70] and of the operation of the endosomal $H^+$/ATPase sensitive to bafilomycin[71,72]. This led to the thought that rotaviruses do not utilize the endocytosis pathway for productive penetration, but rather a direct route through the plasma membrane. However, more recent studies of our group revealed that if bafilomycin A1 is maintained in the medium during entry and replication, it can inhibit infection[73], suggesting the involvement of the clathrin-dependent pathway. On the other hand, another group showed that rotaviruses were able to enter cells where clathrin- or caveolin-mediated endocytosis had been inhibited[74]. Cells treated with methyl-β-cyclodextrin, a drug that sequesters cholesterol from membranes, and cells expressing a dominant-negative mutant of the large GTPase dynamin, which is known to function in several membrane scission events, were not infected by rotaviruses[74]. These findings indicate that cholesterol and dynamin play a role in the entry of rotaviruses, and support the intervention of an endocytic pathway independent of clathrin and caveolin, and perhaps dependent on pH.

#### Permeabilizing Capacity of Outer Layer Proteins

Both pathways of virus entry, the direct penetration and receptor-mediated endocytosis, would entail the transient permeabilization (or disruption) of the plasma or endosomal membrane to permit the translocation of DLP into the cytoplasm. The permeabilizing capacity of TLPs is revealed in conditions that are not likely to be found in the extracellular space.

In fact, purified intact TLPs did not induce membrane permeabilization measured as the leakage of entrapped fluorophore from liposomes and membrane vesicles, or the entry of ethidium bromide into intact cells[62,75,76]. The permeabilizing activity of rotaviruses could be evidenced only when lowering the Ca2+ concentration in the medium below the micromolar level that solubilized the outer proteins, VP4 and VP7. In addition, trypsinization of outer proteins is also required for the effect[33,62,76]. The permeabilizing activity has been associated to VP5*. Recombinant VP5 or its fragments (residues 248 to 474 or 265 to 474) containing an internal hydrophobic domain were able to permeabilize large unilamellar vesicles[77]. Moreover, VP5 seems to mediate size-selective membrane permeabilization, inducing the release of 376-Da carboxyfluorescein (CF), but not 4-kDa fluorescein isothiocyanate-dextran from preloaded liposomes[78]. However, at this point, it is not known whether permeabilization to CF induced by the expressed and purified VP5, and VP5* from a trypsinized and solubilized TLP, are identical phenomena.

On the other hand, expression of these truncated forms of VP5 induced an increase of $Ca^{2+}$ permeability in cell cultures[79]. It was speculated that two discrete domains within VP5 are required for pore formation: an N-terminal basic domain that permits VP5 to associate peripherally with membranes, and an internal hydrophobic domain that is essential for altering membrane permeability[79]. Nevertheless, vesicle permeabilization to CF by exogenous purified VP5 (or VP5 peptides) and permeability to $Ca^{2+}$ of a cell expressing the endogenous VP5 protein may not be necessarily related.

In addition to the effect of VP5*, solubilized peptide(s) of VP7 were also able to permeabilize membrane vesicles loaded with CF. This was observed only when pseudoparticles containing recombinant VP2, VP6, and VP7 (in the absence of VP4) were treated with EGTA to solubilize VP7 and then further hydrolyzed with trypsin[80]. This indicates that VP7 contains a domain sensitive to trypsin, but not accessible to hydrolysis when VP7 is *in situ* on the particle. The permeabilizing effect of the two proteins can be evidenced using purified TLP. In Fig. 2, we can observe that trypsinized TLPs are able to induce CF leakage on addition of EGTA to solubilize the outer capsid proteins. When TLPs grown in a medium containing trypsin are decapsidated in the presence of aprotinin (to protect proteins from contaminant trypsin), a first phase of permeabilization occurs, probably due to the action of solubilized VP5*. A second phase can be obtained by the addition of trypsin in a concentration high enough to overcome the inhibition by aprotinin. This phase may correspond to the effect of VP7 peptides generated by trypsin hydrolysis of the previously solubilized protein. The two phases were observed when the TLPs were retrypsinized *in situ* before outer layer solubilization with EGTA to assert that all VP4 were hydrolyzed into VP5* and VP8*.

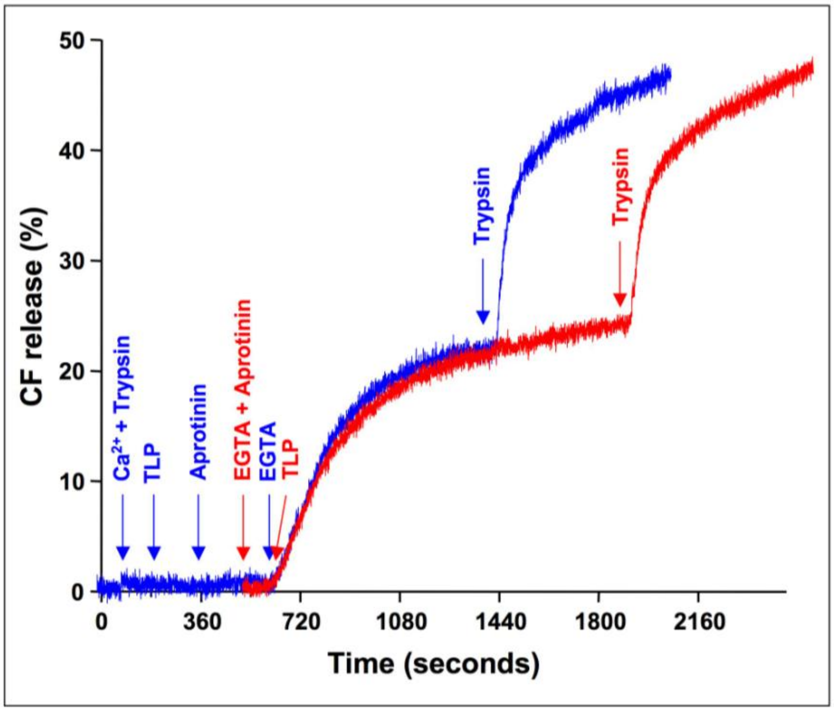

**FIGURE 2**. CF release induced by rotavirus. Purified TLPs grown in the presence of trypsin were added at time zero to a cuvette containing CF-loaded vesicles (70 m*M*) from pig jejunum suspended in standard assay medium (100 m*M* sorbitol and 200 m*M* Tris HCl in 20 m*M* HEPES-10 m*M* Tris [pH 7.4]). *Blue trace*: TLPs were added to the cuvette containing 1 m*M* $Ca^{2+}$ to maintain TLP structure and trypsin (0.01 mg/ml) to hydrolyze VP4 on TLP *in situ*. The action of trypsin was blocked after 5 min by the addition of aprotinin (0.03 mg/ml), and then EGTA (10 m*M*) was added to solubilize the outer layer of TLP and elicit membrane permeabilization. After a plateau was reached, excess trypsin (0.1 mg/ml) was added to hydrolyze the solubilized proteins (VP5*, VP8*, and VP7). *Red trace*: TLPs were added to the cuvette containing aprotinin (0.03 mg/ml) to block any action of contaminant trypsin and EGTA (10 m*M*) to solubilize the outer layer of TLP and elicit membrane permeabilization. After a plateau was reached, excess trypsin (0.1 mg/ml) was added to hydrolyze the solubilized proteins (VP5*, VP8*, and VP7). In both traces, the first phase of permeabilization can be ascribed to the effect of VP5*, whereas the second phase can be attributed to fragments of VP7 and perhaps those of VP5* and/or VP8* (see text). Traces normalized to maximal permeabilization (CF fluorescence) obtained with digitonin (200 μg/ml).

### A Molecular Model for VP4-Membrane Interactions

The molecular interactions between outer layer proteins and the membrane lipids have not been elucidated. However, a model for the architecture of the VP4 spike and rotavirus entry has been proposed, based on the study of the VP4 structure by electron cryomicroscopy, 3D reconstruction, difference map analysis to localize the binding sites for neutralizing monoclonal antibodies, and crystallography of VP4 and VP5*. In this model, VP8* and VP5* mediate receptor binding, membrane permeabilization, and uncoating during transfer across the lipid bilayer[20,21,28].

According to this model, during rotavirus entry, VP4 would transit through three distinct conformations that would correspond to different steps[20]: a first trypsin cleavage between VP8* and VP5* produces a first rearrangement rigidifying the spike by dimeric interactions in the external portion of two VP4 subunits. This interaction would present the VP8* core for cell surface ligand binding, whereas the third VP4 molecule remains flexible. An unknown entry-associated event triggering a second transition to the trimeric conformation has been proposed based on crystal structure. In this transition, disruption of the dimers releases VP8* from VP5* and exposes the hydrophobic domain of VP5*, which may insert into the host cell membrane.

The resulting translocation of the potential membrane-interaction loop towards the foot could disrupt a cellular membrane. This disruption may in turn create a breach that allows a DLP to enter the cytoplasm, or it could lower the local calcium concentration, leading to virion uncoating and subsequent entry events. The foldback transition could also alter interactions with receptors or directly trigger virion uncoating[20]. Although this work represents an important contribution to the understanding of the architecture of the VP4 spike in the particle, its support of a direct entry mechanism through the plasma membrane is rather speculative. However, it is still compatible with an endocytosis model in which $Ca^{2+}$ plays a role in the molecular changes during virus entry, as we detail below.

### A $Ca^{2+}$-Dependent Endocytosis Model

Based on the characteristics of permeability capacities of rotavirus particles and outer layer proteins, a $Ca^{2+}$-dependent endocytosis model has been proposed. This represents an alternative hypothesis for rotavirus entry, where the critical step for virus uncoating and membrane permeabilization is the decrease in $Ca^{2+}$ concentration in the endosome[5,73]. In this model (Fig. 3), the binding of rotavirus to its receptor would induce the formation of an endocytic vesicle, isolating the TLP within an intracellular compartment. A progressive decrease in $Ca^{2+}$ concentration in this vesicle from the extracellular concentration (~1 m*M*) to the intracellular level (~100 n*M*) would occur by simple diffusion through activated $Ca^{2+}$ channels, or by activation of transport mechanisms, as has been reported in other systems[81]. Also, the exposition of VP5 may form a pore and permit $Ca^{2+}$ flux into the cytoplasm[79]. This efflux from the endosome to the cytoplasm seems to be accelerated by the electrical gradient generated by the operation of the vesicular $H^+$ pump sensitive to bafilomycin[73]. This view is further supported by the inhibition of rotavirus entry by increasing the extracellular calcium reservoir by addition of 10 m*M* CaEGTA[73]. It is interesting to point out that neutralizing monoclonal antibodies against VP7 inhibited outer layer solubilization induced by low $Ca^{2+}$ concentration. This effect was not observed with antibodies directed to VP8 or VP5[82]

Once the endosomal $Ca^{2+}$ concentration equilibrates with that of the cytoplasm, below the critical level for stability of the outer capsid, the virus sheds its outer proteins and these in turn lyse the vesicle membrane, permitting the DLP to pass into the cytoplasm[62]. Both VP5* and VP7 would, either by themselves or in concert, act in the destabilization and disruption of the membrane. VP5, upon interaction with the membrane, might induce a breach[20] or a pore[77], favoring $Ca^{2+}$ diffusion from the endosome to the cytoplasm. The involvement of VP7 would imply its hydrolysis by proteases after solubilization. This may be effected by trypsin associated to the rotavirus capsid, and its activation by solubilization of outer capsid proteins or by endosomal proteases[83]. The decrease in $Ca^{2+}$ concentration within the endosomal

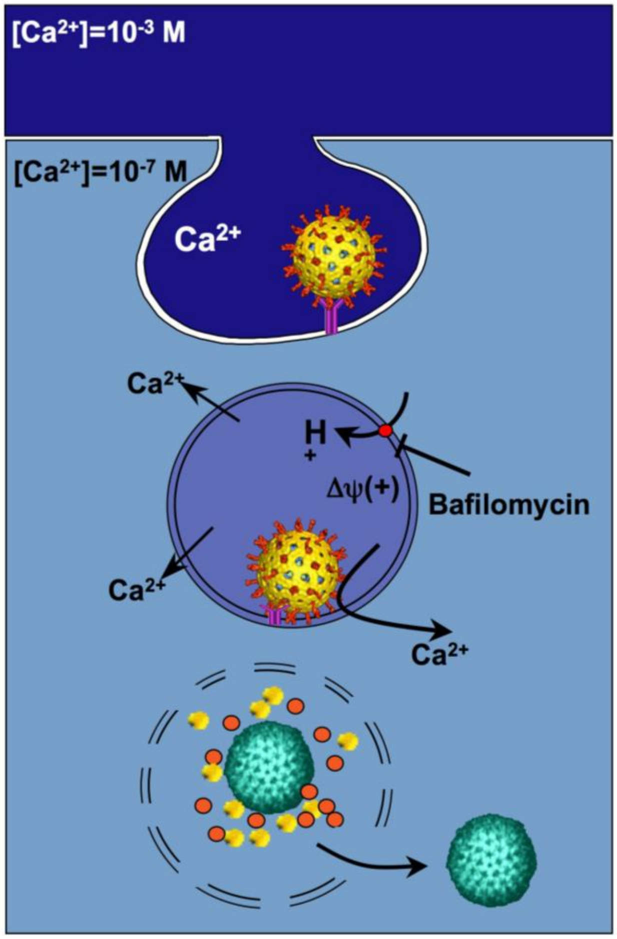

**FIGURE 3**. Hypothetical model of rotavirus entry by endocytosis. After rotavirus particles bind to receptors on the cell surface, they are endocytosed into vesicles together with extracellular fluid containing $Ca^{2+}$ in the 1 m*M* range. Once inside the endocytic vesicle, $Ca^{2+}$ is transported into the cytoplasm driven by the large concentration gradient (from 1 to 100 n*M*). The exposition of VP5 may form a pore and permit $Ca^{2+}$ flux into the cytoplasm. The electrical gradient (positive inside) generated by the v-type $H^+$ pump provides an additional force for $Ca^{2+}$ extrusion out of the endosome. Once $Ca^{2+}$ has dropped to a critical concentration, the virus uncoats and the external solubilized proteins, VP5* and perhaps VP7, permeabilize and lyse the endosomal membrane. In this way, the DLP gains access to the cytoplasm and replication is activated.

vesicle in this model would be equivalent to the decrease in pH for other enveloped and nonenveloped viruses[1]. The $Ca^{2+}$ dependence of the structure of the rotavirus particle, the characteristics of the membrane permeabilization by solubilized outer proteins, and the $Ca^{2+}$ gradients of the cell compartments are fully consistent with this hypothesis.

### ROTAVIRUS ASSEMBLY

Replication of rotaviruses takes place in the cytoplasm with a final stage of morphogenesis within the ER. One could describe three phases of replication: (1) translation and synthesis of viral proteins; (2)

replication of the dsRNA, genome packaging, and DLP assembly; and (3) budding of DLP into the ER for the acquisition of the outer layer. These steps are represented in Figs. 4 and 5. The knowledge on viral replication thus far acquired is based on structural studies, the expression and coexpression of individual viral proteins, silencing of individual genes by siRNAs, and the effects that these manipulations have on the physiology of the infected cell. In particular, the siRNA technique is beginning to provide elements for the dissection of the maze of viral protein interactions in the process of replication and morphogenesis of rotavirus. A summary of these findings is presented in Table 1.

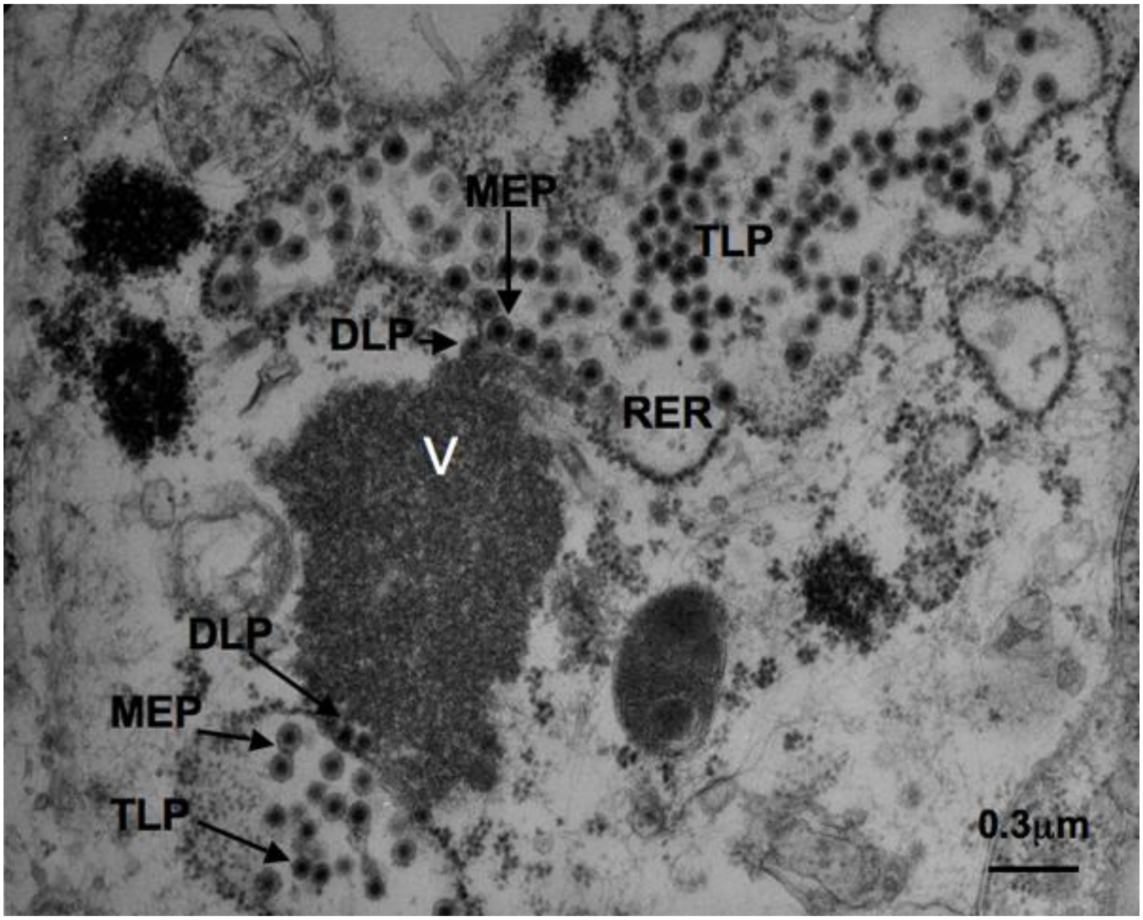

**FIGURE 4**. Ultrastructure of rotavirus-infected MA104 cell. DLPs can be observed emerging from the viroplasm (V) and budding into the RER, acquiring ER membrane. These membrane-enveloped particles (MEP) can be seen near the viroplasm or in proximity to the RER membrane. Towards the center of dilated cisternae, TLPs devoid of enveloping membrane can be observed.

### Translation and Synthesis of Viral Proteins

Once the DLP reaches the cytoplasm after the loss of the outer capsid layer, transcription is activated. This transition may be linked to the low $Ca^{2+}$ concentration in the endosome or cytoplasm as hypothesized above[84]. It is known that the DLPs and no TLPs are the transcriptionally competent subviral particles, producing 11 capped (+) RNAs[85]. The dsRNA segments are transcribed within the structure of the DLP by VP1, which has been identified as an RNA-dependent RNA polymerase (RdRp) and capped by VP3, which has guanyltransferase and methyltransferase activities[86,87,88,89]. The synthesized mRNAs exit the DLP through 12 aqueous channels (type I) that pass through both the VP2 and VP6 layers[90,91].

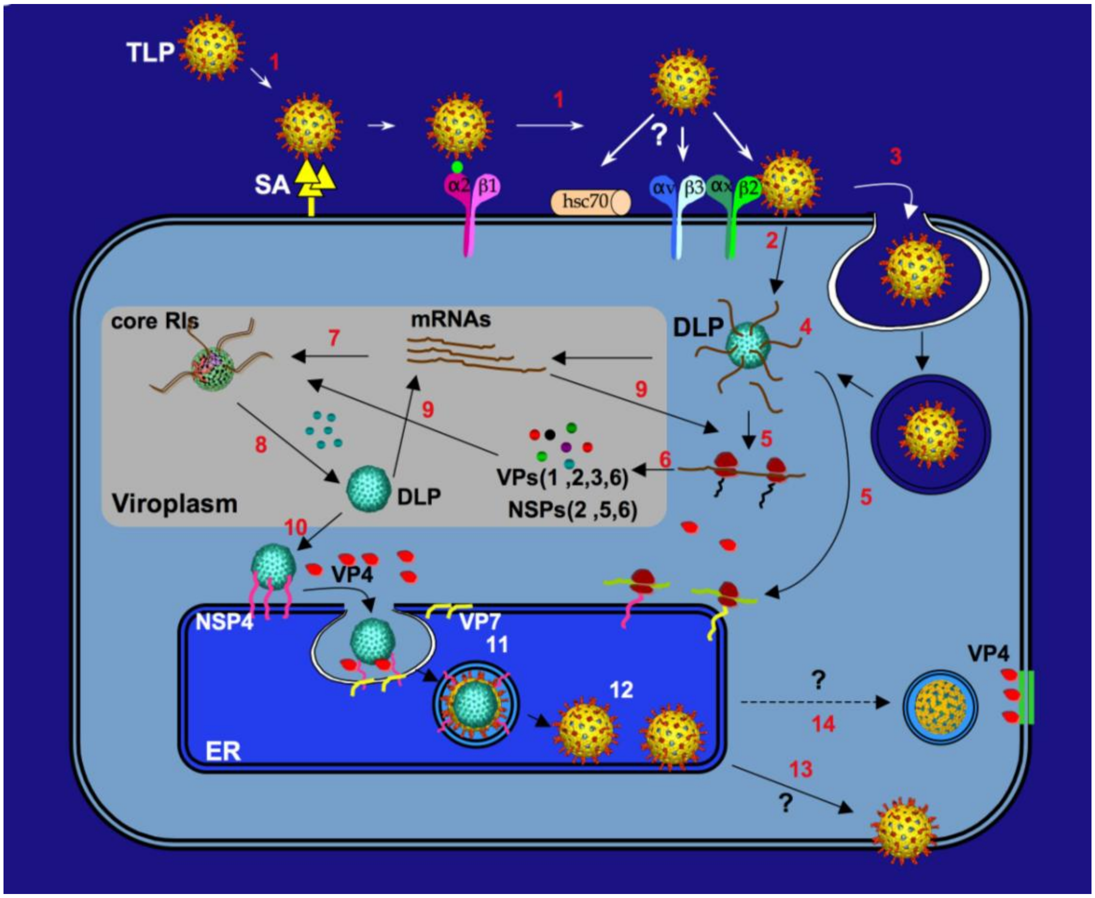

**FIGURE 5**. Schematic representation of the steps of rotavirus replication. (1) Trypsinized TLP binds to one or more receptors on cell surface. (2) Conformational changes on virus permitting penetration by a direct route to the cytoplasm, or (3) by $Ca^{2+}$-dependent endocytosis. (4) Solubilization of the outer protein layer generating DLP that reaches the cytoplasm; activation of transcriptase in DLP and synthesis of mRNA. (5) Translation of mRNA in polysomes and ER-bound ribosomes; synthesis of viral proteins. (6) Accumulation of viral proteins and mRNA, and nucleation of viroplasm. (7) Assembly of core replication intermediates (core RIs) and (–) RNA synthesis. (8) Assembly of DLP. (9) Secondary transcription from DLP and amplification of protein synthesis. (10) Binding of DLP to NSP4 and budding into the ER. (11) Acquisition of the membrane envelope together with VP7, NSP4, and perhaps VP4. (12) Removal of membrane lipids and NSP4, and accumulation of mature TLP. (13) Release of viral progeny through cell lysis or, alternatively, by (14) traffic of immature TLP (without VP4) to rafts containing VP4, and acquisition of VP4 and eventual release at apical membrane without lysis.

The mRNAs are translated in polysomes generating the 12 viral proteins (six structural and six nonstructural) with a concomitant shut-off of cellular protein synthesis. The nonstructural protein NSP3 has been implicated in both processes. Rotavirus mRNAs lack a poly(A) tail, but have instead a consensus sequence at their 3' ends that binds NSP3, acting as a functional homologue of poly(A)-binding protein (PABP). NSP3 also interacts with the cell-initiation factor eIF4GI. It is widely believed that these interactions lead to the translation of rotaviral mRNAs, impairing at the same time the translation of cellular mRNAs[92,93,94]. However, recent reports suggest that NSP3 is neither required for the translation of viral mRNAs nor essential for virus replication in cell culture[95,96]. Using RNA interference to block NSP3 expression in infected cells, it was shown that the synthesis of viral proteins was not decreased and the yield of viral progeny was increased, which correlated with an increased synthesis of viral RNA. Silencing of NSP3 expression re-established the cellular protein synthesis. Therefore, this protein might play a role in the shut-off of cellular protein synthesis[95,96]. Silencing of other viral proteins, such as VP2, NSP2, and NSP5, also restored the synthesis of cellular proteins through an undefined mechanism[96].

## TABLE 1A
## Effect of Silencing the Expression of Individual Viral Proteins on Infectivity, Protein Synthesis, Morphogenesis, and Physiology of Infected Cell

| | siRNA Irrelevant | siRNA VP2 | siRNA VP4 | siRNA VP7 | siRNA NSP1 | siRNA NSP2 | siRNA NSP3 | siRNA NSP4 | siRNA NSP5 |
|---|---|---|---|---|---|---|---|---|---|
| **Infectivity** | 100% | ND | Reduced to 1–25%[131,133,157] | Reduced to 1–30%[98,129,131,132,133] | No effect[98] | Reduced to 10%[98] | Threefold increased[95] | Reduced to 1–25%[129,131,132,133] | Reduced to 20–28%[107] |
| **Particles** | No effect on TLP/DLP relationship (CsCL, PAGE, EM)[98,129,132,157] | ND | Spikeless TLP (CsCL, PAGE, EM)[157] | Reduction of TLP, accumulation of DLP (CsCL, PAGE)[98] | No effect | ND | ND | Reduction of TLP and DLP, accumulation of empty particles (CsCL, PAGE)[129,132] | ND |
| **Protein synthesis** | Shut-off of cellular protein synthesis; redistribution of PABP from cytoplasm to nucleus[96]; phosphorylation of eIF2a[96] | Inhibits synthesis of the other viral proteins, while the cellular protein synthesis was restored[96]; inhibition of phosphorylation of eIF2a[96] | No effect on synthesis of other viral proteins[131]; decreased viral protein association to rafts[133] | No effect on synthesis or distribution of other viral proteins[129, 131]. | Did not affect synthesis of other viral proteins[98] | Inhibits synthesis of the other viral proteins[98]; cellular protein synthesis restored[96]; inhibition of phosphorylation of eIF2a[96] | Synthesis of viral proteins was not decreased, while the cellular protein synthesis was restored[95]; inhibited redistribution of PABP from cytoplasm to nucleus[98] | Affected the intracellular accumulation[129] | Reduced synthesis of viral proteins[107]; cellular protein synthesis restored[96]; inhibition of phosphorylation of eIF2a[96] |
| **Morphogenesis** | VP1, VP2, VP3, VP6, NSP2, NSP5 mRNA, dsRNA in viroplasm associated to ER; DLP assemble in viroplasms and bud into the ER; formation of MEP in ER and maturation to TLP; redistribution of ERGIC 53 from tubulovesicular structure to more diffuse staining in juxtanuclear region[95,98,107,129, 131,132,133,157] | Reduced number and size of viroplasms[96] | Viroplasm in closed apposition to ER; DLP budding, NEVP, Virions (in EM)[157]; normal viroplasm and DLP budding into the ER[133]; accumulation of nonenveloped immature particles within the ER; particles aggregate forming paracrystalline array (EM)[133]; decreased association of TLP to rafts[133] | No effect on viroplasms, no reduction of quantity of other viral proteins[129]; inhibition of outer-capsid morpho-genesis[98]; reduced association of TLP with rafts, reducing the titer to 60 % and quantity of total viral proteins to 30 %, without reduction of quantity of associated viral proteins[133]; accumulation of MEP in ER (EM)[129,133] | No impact on the formation of viroplasms[98] | Inhibits viroplasm formation, genome replication, and virion assembly[98] | ND | Smaller viroplasms (IF)[132]; viroplasms not associated with ER, no budding of DLP (EM); free DLP in the cytoplasm (EM)[129,133]; affected the cellular distribution of several viral proteins[129,132]; reduced the association of TLP with rafts, reducing the titer 30 % and quantity of associated viral proteins to 55%[133] | Reduced number and size of viroplasms and altered intracellular distribution of other viroplasm-associated proteins; reduced synthesis of viral (+)RNA and dsRNA[107] |
| **Physiological and biochemical effects** | Increase of plasma membrane $Ca^{2+}$ permeability, $[Ca^{2+}]$cyto, and $^{45}Ca^{2+}$ pools, and decrease of agonist-releasable $Ca^{2+}$ pools[131]; cytopathic effect[95]; prevents the formation of SGs[96] | ND | No effect on the increase of plasma membrane $Ca^{2+}$ permeability, $[Ca^{2+}]$cyto, and $^{45}Ca^{2+}$ pools, and the decrease of agonist-releasable $Ca^{2+}$ pools[131] | Partially reduced increased $Ca^{2+}$ permeability of plasma membrane, $[Ca^{2+}]$cyto, and $^{45}Ca^{2+}$ pools and the decrease of agonist-releasable $Ca^{2+}$ pools[131] | ND | ND | Delayed cytopathic effect[95] | Inhibited the increase of $Ca^{2+}$ permeability of plasma membrane, $[Ca^{2+}]$cyto, and $^{45}Ca^{2+}$ pools, but not the decrease of agonist-releasable $Ca^{2+}$ pools[131] | ND |

## TABLE 1B
## Effect of Silencing the Expression of Individual Viral Proteins on Expression and Distribution of Viral Proteins in Rotavirus-Infected Cells

| | siRNA Irrelevant | siRNA VP4 | siRNA VP7 | siRNA NSP2 | siRNA NSP4 | siRNA NSP5 |
|---|---|---|---|---|---|---|
| **VP2** | Colocalizes with NSP2 and VP6 in viroplasm[132] | ND | No distribution changes[129] | ND | Colocalizes with NSP2[132] in smaller viroplasms and diffuse in cytoplasm [129,132] | Colocalizes with remnant NSP5 and NSP2 in smaller viroplasms and NSP2 diffuse in cytoplasm[107] |
| **VP4** | Perinuclear ring-like or semicircular structures and a filamentous array distribution[129]; VP4 partially colocalize with ERGIC53 (perinuclear VP4)[107] | ND | No distribution changes (granular, perinuclear, cytoplasm)[129] | ND | Most of the perinuclear structures disappeared, while the filamentous signal of VP4 remained more apparent[129] | Perinuclear ring-like or semicircular structures disappeared leaving a finely punctated pattern; filamentous array distribution is maintained [107] |
| **VP6** | Colocalizes with NSP5 in periphery of viroplasm[132] | ND | Change in the distribution with a large proportion not associated with viroplasms[98]; no distribution changes (granular, perinuclear)[129] | ND | Colocalizes with VP2 in smaller viroplasms and perhaps with diffuse form in cytoplasm[132]; forms filaments that appear to extend to the periphery of the cell instead of being localized to viroplasms[129] | Forms filaments that appear to extend to the periphery of the cell instead of being localized to viroplasms[107] |
| **VP7** | Perinuclear pattern of distribution, probably due to its homogeneous distribution along the ER membrane[107]; distribution of mature VP7 (on TLP) similar to that of VP4: partially colocalize with ERGIC53 (perinuclear) and no colocalization with cis-golgi[133] | ND | ND | ND | More diffuse, although still perinuclear pattern of distribution[129] | More diffuse perinuclear signal[107] |
| **NSP2** | Colocalizes with NSP5 in viroplasm[132]. | ND | ND | ND | Colocalizes with NSP5 in smaller viroplasms and diffuse in cytoplasm [132]. | ND |
| **NSP4** | ND | ND | ND | ND | ND | Perinuclear ring-like or semicircular structures disappeared leaving a more dispersed finely punctated pattern[107] |
| **NSP5** | Colocalizes with NSP2 in viroplasm[132] | No distribution changes[157] | Normal distribution; did not impede viroplasm formation[98] | Diffuse in cytoplasm[98] | Colocalizes with NSP2 in smaller viroplasms and diffuse in cytoplasm [132] | ND |

**Abbreviations to Table 1A and 1B:** MEP, membrane-enveloped particle; SG, stress granule; NEVP, nonenveloped viral particle; ND, not determined; other abbreviations described in text.

### DsRNA Replication and DLP Assembly

Once a critical mass of viral proteins and viral mRNA are synthesized, they accumulate in viroplasms that correspond to discrete, large, electron-dense cytoplasmic inclusions. In these structures, rotavirus protein assembly, viral genome packaging, replication, and the formation of DLP seem to take place[97,98]. In these specialized inclusions, structural and nonstructural proteins (VP1, VP2, VP3, and VP6; NSP2, NSP5, and NSP6) are accumulated[99,100]. Coexpression, localization, and RNA interference experiments have implicated both NSP2 and NSP5 in the nucleation of viroplasms, recruitment of proteins of the core, and virus replication[97,98,101,102,103,104,105,106,107,108,109].

NSP5 is a phosphoprotein rich in Ser and Thr residues, which undergoes O-linked glycosylation[110,111]. Upon interaction with NSP2, NSP5 gets hyperphosphorylated, which would render the protein insoluble, favoring its localization into punctate viroplasm-like structures (VLS)[97,103,105,112,113,114,115]. However, expression of the fusion protein NSP5-EGFP led to the formation of VLS without the intervention of NSP2. It might be possible that the EGFP moiety reproduced conformational changes elicited by NSP2 to precipitate NSP5-EGFP and form VLS. Interestingly, this phenomenon is calcium regulated[116]. Therefore, viroplasm formation may be dependent on the changes in cytoplasmic $Ca^{2+}$ concentration induced by infection[5,117]. NSP2 is a 35-kDa basic protein that exhibits nucleoside triphosphatase activity (NTPase) and affinity for ssRNA[118]. These properties have been suggested to play a role in genome packaging.

Viroplasms are the putative sites of core and DLP assembly (Fig. 4), and RNA replication (minus-strand synthesis). The current view is that the first replication intermediate (precore RI) is formed by one copy of VP1 (RdRp) and VP3 each, associated with one segment of (+) mRNA. Each precore RI associates with five dimers of VP2, one octamer of NSP2, and a dimer of NSP5, forming the core replication intermediate. In turn, the core would self-assemble by the polymerization of 12 of these complexes[90,119,120,121]. The self-assembly capacity of VP2 has been observed with the expression of VP2 alone, resulting in the production of particles called pseudocores that have the same geometry as viral cores[122]. The (+) RNAs synthesized by the DLP in the viroplasm serve as templates for the synthesis of the (–) RNAs to generate dsRNA genome segments. The dsRNA replication takes place at the same time as the packaging of the 11 genome segments, produced at equimolar levels, into newly formed cores[123,124]. The question remains as to how a virus with a segmented genome can integrate a single copy of each one of those segments into its particle. Concurrently with dsRNA synthesis, the VP6 layer assembles on the core to form DLP[90,121]. It has been shown that VP6 expressed alone in the baculovirus system assembles in trimers and tubules[125,126,127]. However, interaction of VP6 with VP2 on the core leads to the formation of chimeric, empty, DLP (VLP2,6)[128]. In the viroplasm of infected cells, the acquisition of the VP6 layer is thought to depend on the previous assembly of the core.

Viroplasms are dynamic structures, topographically heterogeneous. The interior domain contains VP1 and VP2, and the nonstructural proteins NSP2 and NSP5. This area is also the site of synthesis of (+) RNA, consistent with being the site of progeny core formation and genome packaging into the single-layer particles[102]. The exterior domain is rich in VP6, most probably unassembled, since the amount of DLP seen by electron microscopy (EM) in the periphery of the viroplasm is rather small. The exterior domain also contains NSP4, which may play a function in recruiting unassembled VP6 for the morphogenesis of cores to DLP[102,129,130].

Silencing the expression of NSP4 causes modifications in the replication and morphogenesis of rotaviruses, including profound changes in the distribution of VP6 from viroplasm to cytoplasm and aggregation into filamentous arrays; a severe reduction in the numbers of viroplasms, DLP, and TLP; and a loss of the spatial relationship of the viroplasm with the ER (Table 1)[129,131,132,133]. Therefore, NSP4 as an integral protein of the ER may organize the elements of the viroplasm and the generation of DLP, as well as the entry of DLP into the ER for the acquisition of the outer protein layer of the capsid (see below).

### Morphogenesis of Viral Particles in the Endoplasmic Reticulum

A particular characteristic of rotavirus morphogenesis is the maturation of the particles in the ER, one of the main cell calcium reservoirs. The stability of the mature TLP structure is not compatible with the low calcium concentration found in the cytoplasm[33,134]. Therefore, one may think that the rotavirus takes advantage of the high calcium concentration of the ER for the acquisition of the outer protein layer. This is a complex process involving numerous steps.

The DLP subviral particles assembled in viroplasms bind to the ER to bud into this compartment (Fig. 4). NSP4 acts as a receptor for the DLP[135,136,137]. Its cytoplasmic C-terminal domain interacts with VP6 on DLP[138,139,140]. During the budding process, DLPs acquire a transitory membrane envelope derived from the ER (Fig. 4). These membrane-enveloped particles (MEP) contain NSP4 and VP7, which are integral membrane proteins in the ER[141]. Then, the particle matures by a selective retention of the external capsid protein VP7 and perhaps VP4 (see below), and the elimination of NSP4 and the membrane lipids.

Based on an accumulated body of evidence, the picture of the final assembly of rotavirus in the ER begins to be clarified. The interplay of at least three factors appears to direct this process: the folding of VP7 and NSP4, the ER $Ca^{2+}$ concentration, and the function of ER chaperones.

The high $Ca^{2+}$ concentration in the ER is important not only for the assembly of the external layer of the capsid, but also for the removal of the transient envelope. Dissipation of the ER $Ca^{2+}$ gradient during infection affects the maturation of rotavirus, stopping the process at the MEP stage[142,143]. In these conditions, VP7 is excluded from the heteroligomeric complexes of NSP4, VP7, and VP4[143], and it is not recognized by conformation-specific monoclonal antibodies[142]. Furthermore, it has been shown that VP7 that was expressed by a recombinant herpes simplex virus-1, or contained in purified rotavirus particles, lost reactivity with a neutralizing monoclonal antibody upon chelation of calcium by EGTA[31]. Immunoelectron microscopy experiments in infected MA104 cells suggest that, in the absence of $Ca^{2+}$, VP7 did not assemble onto virus particles and remained in the cytoplasm outside the ER[144]. The significance of this finding remains to be assessed. During infection, calcium depletion in the ER impaired the N-glycosylation of VP7 and NSP4 without inhibiting viral protein synthesis. In addition, synthesized VP7 appeared to be misfolded, since it was not recognized by conformation-specific antibodies[142]. These effects are somewhat similar to those of tunicamycin, a glycosylation inhibitor, suggesting that the first event of replication affected by ER $Ca^{2+}$ emptying may be the glycosylation of VP7 and NSP4[145,146]. However, glycosylation of VP7 does not seem to be essential for TLP assembly, since the SA11 clone 28 where VP7 is not glycosylated undergoes normal morphogenesis producing a high virus yield[147,148].

The following steps to produce mature rotavirus particles are poorly known, and involve the removal of NSP4 and lipids from the membrane-enveloped subviral particles. This process seems to be directed by VP7 since the silencing of this protein or prolonged 1,4-dithiothreitol treatment (DTT), which affects disulfide bond formation and VP7 folding, did not block the budding of DLP into the ER, but arrested maturation at the MEP stage in this compartment[129,133,149]. However, NSP4, which has a destabilizing activity on liposomes and microsomes, might play a role in the removal of the transient membrane envelope[150]. These steps might also be related to the intervention of the $Ca^{2+}$-dependent ER chaperones and oligosaccharide residues in the correct folding of VP7 and NSP4[151,152,153]. Glycosylated NSP4 seems to interact with calnexin and with protein disulfide isomerase (PDI) as a chaperone. On the other hand, VP7 does not interact with calnexin, but its correct folding is dependent on the enzymatic action of PDI. Interestingly, rotavirus infection or the expression of NSP4 alone induced the up-regulation of BiP (GRP78) and endoplasmin (GRP94), two ER resident glucose-regulated proteins[154]. Recent studies where the expression of ER chaperones was silenced show that GRP78, PDI, calnexin, calreticulin, but not GRP94 or ERP57, caused a reduction in the yield of infectious virus of about 50%[155]. These results suggest that these chaperones are involved in the quality control of rotavirus morphogenesis.

In conclusion of this point, it is tempting to propose that any condition that impairs the proper folding of VP7 would exclude the protein from being assembled onto the DPL and hence cause the lack of removal of the lipid membrane. Particles inside MEP in this case would resemble DLP with VP4 attached, but not VP7. The events that lead to selective removal of lipids remain to be investigated.

A recent report has shown that the expressed NSP4-EGFP or NSP4 in infected cells was initially localized in the ER, but later associated to a vesicular compartment throughout the cytoplasm. NSP4-EGFP or NSP4 in this compartment did not colocalize with ER, ERGIC, Golgi, endosomal, or lysosomal markers but colocalized with the autophagosomal marker LC3 and was dependent on intracellular calcium levels[156]. The role of this compartment, and the fate and function of NSP4 associated to it, is not known.

The how and where the spike protein VP4, a cytoplasmic synthesized protein, is assembled onto the viral particle is a matter of debate. The classical view is that VP4 binds to the DLP as it buds into the ER. However, VP4 did not seem to be essential in this process since the silencing of VP4 expression did not inhibit DLP budding into the ER or lipid membrane removal, and led to the formation of spikeless TLPs[157]. The hypothesis of the acquisition of VP4 on the particle in the ER is supported by a series of evidence: (1) VP4 has been detected on TLPs and permeabilized MEP in the ER by immunoelectron microscopy[158]; (2) the C-terminal domain of NSP4 has a binding site for VP4[159,160]; (3) VP4 can form heteroligomeric complexes with NSP4 and VP7, and it is found in MEP[143]; (4) there is extensive colocalization between the ER proteins, NSP4 and VP7, and VP4[130]. On the other hand, an alternative model of rotavirus assembly that utilizes an untypical trafficking pathway has been proposed[161,162,163,164]. In this case, VP4 would assemble after VP7. Once VP7-coated particles leave the ER, they would be transported to the apical cell surface interacting with cytoplasmic VP4 in lipid rafts bound to cytoskeletal elements and in transit to the plasma membrane for an apical release without cell lysis[162,165,166]. However, a number of results are not consistent with the hypothesis that VP4 assembles after VP7 in a post-ER cytoplasmic compartment: (1) electron cryomicroscopy reconstructions show a VP4 domain buried beneath the VP7 layer that has to interact with VP6 before acquiring VP7[17,19]; (2) in *in vitro* recoating experiments, it was evidenced that VP4 has to assemble onto DLP before VP7 to reconstitute infectious rotavirus particles[36]; (3) when VP4 was silenced by siRNA, viral particles accumulated in the ER of infected cells were organized in unusual paracrystalline arrays, indicating that the absence of VP4 alters TLP structure inside the ER[133]. Nevertheless, the mechanisms of rotavirus assembly might differ in polarized intestinal-like cells.

The high $Ca^{2+}$ concentration inside the ER is also required for the stabilization of already mature viral particles accumulated in this compartment. $Ca^{2+}$ depletion of the ER at the end of the infection period disassembled the outer layer inside the ER, inducing a reduction of infectious TLPs[134]. The loss of infectious capacity suggests that the viral particles in this compartment are mature and infectious, already containing VP7 and VP4.

### VIRUS INFECTION AND CA$^{2+}$ HOMEOSTASIS

In addition, infection by itself induces changes in calcium homeostasis of the cell that may be advantageous to virus replication[117,142]. Among these perturbations, we have measured a progressive increase in plasma membrane $Ca^{2+}$ permeability, which leads to an elevation of cytosolic $Ca^{2+}$ concentration and enhancement of sequestered $Ca^{2+}$ pools in the ER[5,117,142]. This effect is likely due to the activation of SERCA pumps (Fig. 6).

NSP4 appears to be responsible for the many effects of infection on $Ca^{2+}$ homeostasis. The intracellular expression of NSP4-EGFP fusion protein in mammalian cells elevates basal intracellular calcium levels[148,167] and induces a large increase in plasma membrane $Ca^{2+}$ permeability similar to that brought about by infection[148].

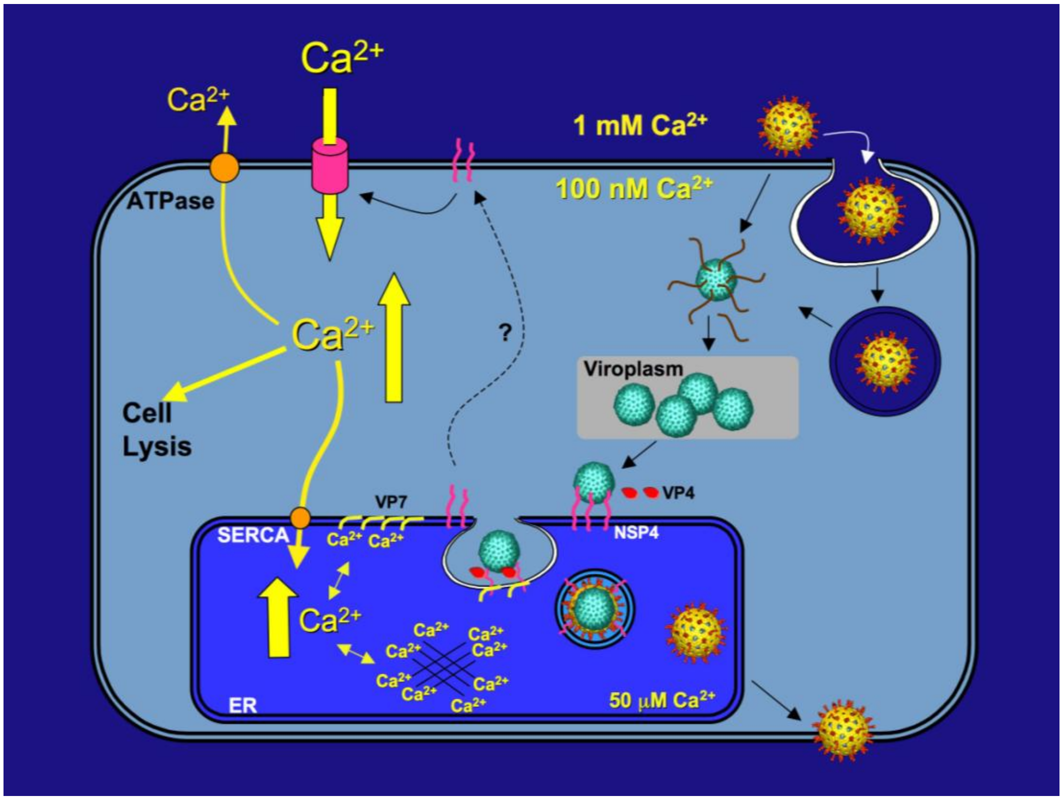

**FIGURE 6.** Changes in $Ca^{2+}$ homeostasis induced by rotavirus infection. It is hypothesized that NSP4 synthesized in the ER travels to the plasma membrane to form a channel or activate a cellular $Ca^{2+}$ pathway. This induces a progressive increase in plasma membrane $Ca^{2+}$ permeability, which leads to an elevation of cytosolic $Ca^{2+}$ concentration and enhancement of sequestered $Ca^{2+}$ pools in the ER. This effect is likely due to the activation of SERCA pumps. $Ca^{2+}$ may be buffered in the ER by chaperones and viral proteins (VP7), reducing the free $Ca^{2+}$ pools releasable by agonists. The elevation of cytosolic $Ca^{2+}$ concentration may be responsible for cell death induced by infection.

Rotavirus infection also leads to a progressive depletion of agonist-releasable ER pools. However, this effect does not seem to lead to ER $Ca^{2+}$ depletion since the $^{45}Ca^{2+}$ uptake is increased[168]. Taking another approach, it was recently shown that the silencing of VP7 expression partially inhibited the increase of $Ca^{2+}$ permeability in infected cells, whereas the silencing of NSP4 completely blocked this effect[131]. However, the silencing of VP7 also reduced the synthesis of NSP4 in Cos 7 cells and impaired the assembly of viral particles within the ER[131]. This may suggest that reduction of $Ca^{2+}$ permeability in VP7-silenced cells is caused by the reduction of NSP4 synthesis.

Therefore, NSP4 expressed during infection appears to be responsible for the changes in $Ca^{2+}$ homeostasis. NSP4 may increase $Ca^{2+}$ permeability directly by forming a plasma membrane $Ca^{2+}$ channel. The viral protein would traffic from the ER to the plasma membrane, where this channel would become active by unknown mechanisms such as conformational changes, oligomerization, and/or proteolysis. Supporting this view, a truncated form of recombinant NSP4 expressed in Vero cells corresponding to the transmembrane segment of the molecule (aa 1–89) was able to escape the ER via a brefeldin A–sensitive pathway and reach the plasma membrane[169]. On the other hand, the full-length, glycosylated NSP4 molecule has been recently detected in plasma membrane rafts interacting with caveolin, in rotavirus-infected or NSP4-EGFP–expressing cells[170]. In this case, the trafficking of NSP4 involved a Golgi-bypassing transport as judged from its endo-H sensitivity. However, the inhibitory effect of brefeldin A on the increase of permeability elicited by infection supports the involvement of a Golgi-dependent pathway[168]. It is interesting to note that the full-length NSP4 molecule was secreted via a brefeldin A–

sensitive pathway in Caco2 cells[171]. Whether NSP4 secretion, localization at the plasma membrane, and the increase in $Ca^{2+}$ permeability are related phenomena remains to be investigated.

Within our hypothesis, NSP4 would be acting as a viroporin[172]. These viral proteins have a hydrophobic transmembrane domain that interacts with the lipid bilayer, forming hydrophilic pores by oligomerization, giving rise to enhanced passage of ions and small molecules[172,173]. NSP4 has a hydrophobic domain that spans the membrane and it has been shown to undergo oligomerization under certain conditions[160,174,175]. The topology of NSP4 at a putative site in the plasma membrane remains to be elucidated. The mechanisms of $Ca^{2+}$ passage induced by this protein in rotavirus-infected cells are currently under investigation.

### CONCLUDING REMARKS

Although the last 10 years have seen remarkable progress in the understanding of the rotavirus cycle, thanks to the application of new potent techniques, a number of important questions still remain unanswered. The binding to the membrane seems to be a multistep process, with the participation of sequential or parallel-specific receptor-viral protein interactions. However, the succession of events that leads to virus entry is not yet known, nor the route of productive penetration. Evidence for the $Ca^{2+}$ dependent of endocytosis model of entry, as well as for the direct pathway model, is still insufficient and, at most, indirect. The dynamics and macromolecular organization of the viroplasm and the assembly of the core need more studies. The mechanism by which a virus with a segmented genome can integrate a single copy of each one of the segments into its particle is particularly intriguing. At this point, the site of the acquisition of VP4 on the outer layer of TLP remains controversial. The role of NSP4 in coordinating viroplasm structure, DLP budding into the ER, and TLP maturation appears pivotal to rotavirus replication and assembly. Equally, the participation of NSP4 in $Ca^{2+}$ homeostasis changes during infection and pathogenesis is still of utmost interest. Is NSP4 a viroporin and a virotoxin? Another unsolved theme is the mechanism of viral progeny release and cell death. Finally, the achievement of a reverse genetics system for rotavirus promises to provide light on the many obscure aspects of the rotavirus cycle and pathogenesis[176].

### ACKNOWLEDGMENTS

The authors wish to thank Maria Elena Chemello for constructive comments on the manuscript. Work in the authors' laboratory is supported by LOCTI program financed by TOTAL Venezuela S.A., Helmerich Paine Venezuela, and Laboratorios Chacao.
